# Cases of Immobility of the Inferior Maxillary Bone, Produced by the Abuse of Mercury

**Published:** 1853-04

**Authors:** H. H. Toland

**Affiliations:** San Francisco, California.


### ARTICLE XXI.

*Cases of Immobility of the Inferior Maxillary Bone, produced by the abuse of Mercury.* By H. H. Toland, M. D., of San Francisco, California.

The belief that mercury is a specific for every variety of fever, no difference what may be the violence, stage, or type, if administered until ptyalism is produced, is, unfortunately, too prevalent amongst the physicians of the Middle and Southern States. Calomel is, unquestionably, a very valuable remedy, if administered as a cathartic, both in intermittent and remittent fevers; but to give from two to five grains, every two or three hours, as is the practice of many, until the fever either yields, or ptyalism is produced, is, to say the least, in the fevers above specified, not only injurious, but highly dangerous. No difference how much is given, the patient cannot be salivated, in violent cases, until the fever subsides; it is, therefore, an indication of returning health, and not the cause.

The proximate cause of fever is, by many, considered to be a derangement of the biliary organs, and, consequently, can only be removed by such remedies as have a specific action on the hepatic system. Some years ago, in the upper and middle districts of South Carolina, a fever of a typhoid character from the commencement was not believed to exist, by many even of the most eminent practitioners; and when, during the progress of the disease, typhoid symptoms became so prominent that the most careless observer could not be mistaken in their character, it was considered a bilious fever, that had assumed a typhoid form, and, being violent, the necessity for the administration of calomel was only increased.

Typhoid fever, of a violent form, prevailed extensively in Newberry District during the spring and summer of 1834, (and, also, in Edgefield and Lancaster, as I have since learned from Drs. Burt and Wylie, who not only recognized the disease, but treated it very successfully.) It never terminated under fifteen days, under the most favorable circumstances, viz. by depletion, both general and local, at the commencement, should the pulse be strong and frequent, accompanied with local determinations, indicated by the pain in the head or abdomen, followed by counter-irritants; the mildest laxatives, when required; anodynes, if necessary to procure sleep, or check diarrhea; mucilaginous drinks, and the most simple diet. If the violence of the attack was not moderated during the first two or three days, the fever often lasted from twenty to sixty days, and often terminated fatally, in consequence of extensive ulceration of the mucous membrane of the intestinal canal. But, if treated as it was generally at that period, and as I have too often witnessed in the country, by emetics, drastic cathartics, followed by two grain doses of calomel in combination with tart. antimony every two hours, it almost invariably terminated fatally in eight or ten days; or, sloughing of the mucous membrane of the mouth resulted, which was more distressing and dangerous than the fever for which it was administered.

The same fever still prevails in the middle and upper districts of South Carolina, and is usually called red tongue fever; and many of the physicians have learned, by sad experience, that calomel is not to be relied on for its cure, although some of them still transcend the bounds of moderation and prudence in its administration, as the number of cases resulting from its abuse, that have fallen under my care, abundantly testify. Some die soon after the sloughing process commences; others are unfortunate enough to live, and drag out a miserable and loathsome existence, without the possibility of relief being afforded them by the most skillful surgeon; whilst there are a few amenable to treatment, and to this class I will devote the remainder of this communication.

The above remarks respecting the character of the prevalent fever of the state may be considered irrelevant to the subject under consideration: but, when it is recollected that it is a febrile disease; that the largest quantities of calomel are administered, and its poisonous effects most frequently produced; and also, that in many of the cases of fever, to which the physician is called, calomel, even in the smallest doses, is not only unnecessary, but injurious, I believe they will not be considered as altogether misplaced.

I cannot conceive of a human being, who is not deprived of the sense of vision, being in a worse condition than one who, from the excessive use of mercury, has lost the use of the inferior maxillary bone. When the mucous membrane, as well as the cellular substance, muscles of the mouth and cheeks, slough from its use, the ulcer, after some weeks, cicatrizes; but, instead of, as in ulcerations from any other cause, a membrane, similar to the original, being formed, a strong, dense and unyielding, fibrous substance supplies the place of the mucous membrane and bone. It resembles, somewhat, the cicatrix from a burn, but is more dense and unyielding, so much so, that the mouth is so firmly closed, that, if the teeth and alveolar processes have not been lost by the same cause, it becomes necessary to extract a tooth to prevent starvation; and, should vomiting occur, the patient is in great danger of suffocation in consequence of the contents of the stomach filling the nasal cavity and preventing respiration. The teeth, from want of use as well as the morbid salivary secretion, resulting from the specific inflammation of the salivary glands, become incrusted with tartar, which separates the gums from the teeth, and they are frequently lost, although sound, even after the motion of the jaw has been restored by an operation. So that, under the most favorable circumstances, the patient that is unfortunate enough to fall into the hands of a physician, who believes that calomel is the only curative agent in fever, is liable to all the inconveniences resulting from diseased gums, decayed teeth, morbid salivary secretion, and the offensive exhalations to which they give rise, as well as the derangement of the general health inseparable from continual pain and imperfect mastication.

Case 1.—Being at Newberry village in March, 1836, I was requsted by one of the physicians of that place to operate on a boy, aged twelve years, who, for more than a year, had suffered from immobility of the inferior maxillary bone, accompanied with an opening in the left side of the cheek, about half an inch in diameter, situated the same distance from the angle of the mouth. The cicatrix, for more than an inch around the opening, was thin and exceedingly dense, almost devoid of elasticity, and adhered firmly to both the superior and inferior maxillary bones, by which the motions of the latter were destroyed. The opening in the cheek being lower than the mouth, the saliva flowed continually through it, which rendered his situation much more unpleasant.

The necessary preparations being made, that portion that intervened between the angle of the mouth and the artificial opening, was first divided, and the cicatrix detached extensively, both from the superior and inferior maxillary bones; several teeth, that were loose and covered with tartar, were also extracted. The incisions, by which the edges of the opening were removed, extended more than inch beyond its posterior edge, and included a sound portion of the cheek, so that the wound might be closed with more facility, and present less deformity. In consequence of the entire want of elasticity in the parts implicated in the operation, it was impossible to bring the edges of the wound in direct apposition, until a portion of the indurated substance, both above and below the opening—the shape of the letter V—half an inch wide at the base, and an inch long, was removed. The wound being closed by the interrupted suture, it was found necessary, in order to bring every portion of the other in direct apposition, to insert at least a dozen ligatures, in consequence both of their thinness and unyielding character. A narrow strip of adhesive plaster was applied between each point of suture, and the parts kept as motionless as was consistent with the prehension of aliment. The edges of the wound being exceedingly firm, ulceration, from the pressure of the ligatures, did not take place for ten days; when they were removed, the opening was entirely closed. The proper means being resorted to, after the cicatrix had acquired sufficient firmness, the use of the jaw was restored, and less deformity resulted than would have been supposed from the nature of the difficulty.

Case II.—In September, 1840, Miss B., of Columbia, S. C., was placed under my care. She had been unable to move the lower jaw for more than a year, produced by a sloughing of the mucous membrane of the left cheek, resulting from the abuse of calomel, during an attack of yellow fever, from which she suffered in Augusta, Georgia. She had lost two of the incisor teeth, with the alveolar processes of the right side, which enabled her to take sufficient nourishment to sustain life. Having determined to make an effort for her relief, without an external incision, a long, probe-pointed bistoury with a cutting edge an inch long (the edge, from the remainder of the blade next the handle, having been ground off) was passed between the teeth and cicatrix, and the latter divided superiorly, by pressing the edge of the bistoury forcibly against it; as much of the ligamentous band as possible was then dissected from its connections from above downwards as low as its attachment to the inferior maxillary bone, with the same instrument. The finger was then passed between the teeth and cheek without difficulty, and the remaining portion of the cicatrix was divided transversely, in at least a dozen places, with a small scalpel. The lever was then applied, its branches being covered with buckskin, to prevent injury to the teeth, and the mouth was opened without difficulty. The teeth of the effected side were all loose and covered with tartar; the gums having either sloughed, or been detached by the deposition on the teeth, which were removed, and chloride of soda, properly diluted, directed as a wash. On the third day the lever was again applied, and its use continued every day afterwards for six weeks, when the necessity for its continuance ceased; the wound having healed entirely, and no difficulty being experienced in separating the front teeth, nearly an inch, by the action of the muscles. Great care was necessary during the treatment of this case, in the use of the lever, as all the teeth were somewhat loose, from disease both of the gums and alveolar processes. The tartar having been removed, was kept from accumulating by the use of the tooth-brush and cold water, which is all that is necessary in any case to effect that object.

Case III.—A. G., aged twenty-five years, was operated upon, March 2d, 1841. He had taken, some years before, during an attack of fever in Cheraw, a large quantity of calomel, which produced sloughing of the internal side of the right cheek, from which immobility of the lower jaw resulted; the fibrous band was not more than half an inch wide, and no difficulty was experienced, either in removing it with the bistoury and scalpel, as in the former case, or in opening the mouth with the lever. In the use of this instrument, when considerable force was required to overcome the resistance, the finger was introduced, and, if the division had not been completed, transverse incisions were made until the resistance was entirely overcome, which can be done much more easily and effectually, when rendered tense by the action of the instrument. In this case, the lever was used only a few times, being rendered unnecessary by the patient wearing a cork between his teeth several hours every day, whilst attending to his usual business.

Case IV.—Miss G——, aged seventeen years, of Chester district, S. C., visited Columbia in March, 1850, for the purpose of submitting to an operation, if I thought it advisable. Her situation was particularly distressing, the mouth being so firmly closed that, although she had lost a tooth from the opposite side, it was with the greatest difficulty that she could take a quantity of food sufficient to sustain life; the whole day was required to enable her to take a sufficiency to appease the cravings of hunger, and, on several occasions, she narrowly escaped death from suffocation, in consequence of the contents of the stomach, during an effort to vomit, not finding egress from the mouth, having closed the nasal cavity, and thereby preventing respiration. Of a recurrence of this difficulty, she lived in constant dread. About eighteen months before visiting Columbia, she had taken large doses of calomel for the cure of fever, which produced sloughing of the greater portion of the left side of the face, which, to the astonishment of her friends, in a few months was gradually replaced, and finally closed, except a portion at the angle of the mouth about half an inch wide. When I examined her, the cicatrix extended from the mouth to the ear, and from the molar to the inferior maxillary bone. The corner of the mouth was defective; a portion of the under lip being destroyed, caused the upper one to appear large, and to project over the other.

After a careful examination of the case, although I was unable to determine its precise character, in consequence of the immobility of the inferior maxillary bone, and the projection of the upper teeth, resulting, as I supposed, from the unyielding character of the cicatrix, I determined to make an effort not only to remove the deformity, but also to open the mouth, upon which her future comfort, as well as existence, seemed to depend. The necessary instruments for such operations being prepared, it was performed, in presence and by the assistance, of Drs. Reynolds, Kennedy and Gibson, as well as several medical students. Having administered chloroform, I commenced at the edge of the cicatrix by endeavoring to remove or overcome the resistance at the angle of the mouth, hoping that when the external resistance was removed, the teeth might be so far separated as to allow the introduction of the lever, and, thereby, obtain its assistance during the subsequent part of the operation. When the scalpel was passed under the edge of the cicatrix it came in contact with bone, and, upon extending the dissection, it was apparent that the parts adhered to a solid and smooth surface of bone, which seemed to be extensive, and appeared to be the cause of the immobility of the jaw. Not being provided with a saw, the operation was suspended until one could be procured. The chloroform being again administered, an incision was made, extending from the ear to the angle of the mouth through the cicatrix. Both the superior and inferior portions of the soft parts being dissected up, a smooth and solid bone was exposed, extending from the superior to the inferior maxillary bones, to which it was connected, and from the first molar tooth to the angle of the jaw. This was divided from before backwards, with a small amputating saw; was solid, and, in some portions, half an inch thick. As soon as the bone was divided, the inferior maxillary bone moved with the greatest facility. A portion of the mucous membrane of the upper lip was removed, and the under lip separated from its connection with the newly formed bone. The whole extent of the incision was brought together by a great many points of the interrupted suture, and in such a manner, that the deficiency at the angle of the mouth, as well as the disproportion in the size of the lips, entirely disappeared. A strip of adhesive plaster was applied between each suture, and the most perfect rest enjoined. By the sixth day, upon removing half of the sutures, it was ascertained that union was completed, although the remainder were left two days longer. She left Columbia for the country the tenth day after the operation, and was afterwards treated by Dr. Gibson, of Fairfield, who, as soon as the wound had firmly united, used the lever every day for some months. The newly formed bone came away several months after the operation, leaving the teeth in their original position. One year after this operation, the patient experienced no difficulty in mastication, and was in the enjoyment of good health.

In this case, an extraordinary curative effort was made, and it was only by the formation of bony matter that the deficiency in the cheek could have been restored. The newly-formed bone was covered by a dense, firm and inelastic substance, resembling the cicatrix from a burn, about the eighth of an inch thick, except at its anterior edge. Lint was introduced between the bone and soft parts, after the latter were brought in apposition, to prevent adhesion, and also with the hope that if the connection with the external covering was destroyed, the bone might be separated from its connections by the absorbents; which did occur, and in a much shorter time than was anticipated.

Case V.—A gentleman from the Fork, Richland district, came with his daughter, aged eight years, to Columbia, in August, 1850, with immobility of the lower jaw, resulting from the abuse of calomel. The ligamentous band was extensive and unyielding, and had existed two years.

The operation was similar to that performed for the relief of the second case. Her father having another child sick at home, and believing that he could prevent a return of the difficulty by the use of the instrument, one was furnished him. In a few months it was returned, and the case reported as perfectly cured.

Case VI.—A boy, aged twelve years, from Chester district, was operated upon about the same time, and the cure was progressing rapidly, when he met with a friend of his father from the same neighborhood, and returned home without my consent, for the purpose of avoiding the pain necessarily inflicted by the use of the instrument. I was afterwards told by the physician of the family that the disease returned. He derived no benefit from a mere division of the cicatrix, which shows the great importance of the subsequent treatment.

Case VII.—A gentleman, from Abbeville district, was operated upon in October of the same year. Being unable to leave his business during the time necessary to complete the cure, he returned home in ten days, and took with him an instrument, intending to procure the services of his family physician during the time it might be necessary to continue the treatment. From him I have not heard since, and if he was not cured, it was for the want of proper management.

Three of the above cases had been operated upon previously, viz. the third, fifth and seventh, without receiving any permanent benefit. A mere division of the cicatrix will never avail anything; it should be removed as near to the healthy parts as possible, and what remains, divided by a number of transverse incisions, so as to overcome all resistance, and then, by using the lever every day, the transverse wounds will heal by granulations and effectually prevent a return of the previous difficulty.

I am induced, from the number of cases that have fallen under my notice, during the year 1850, to infer that the practice of administering too much calomel is still common amongst physicians, and that an exceedingly valuable, but dangerous medicine, is still given too indiscriminately in febrile cases. They should bear in mind, that every remedy that is capable of doing much good, is also equally liable, if improperly used, to do great injury; and, that it is, in typhoid cases, most dangerous, and never can be beneficial, which is well known to all who understand the pathology of that disease.

If these remarks induce one practitioner to withhold a dose of calomel in that fever, when it would have been otherwise administered, this object will not have been entirely defeated, and this publication altogether valueless—*Charleston Med. Journ. and Review.*

